# Throwing the dice for the diagnosis of vaginal complaints?

**DOI:** 10.1186/1476-0711-5-4

**Published:** 2006-02-17

**Authors:** Andreas Schwiertz, David Taras, Kerstin Rusch, Volker Rusch

**Affiliations:** 1 Institute for microecology, Herborn, Germany; 2 Institute of Animal Nutrition, Faculty of Veterinary Medicine, Free University Berlin, Germany

## Abstract

**Backround:**

Vaginitis is among the most common conditions women are seeking medical care for. Although these infections can easily be treated, the relapse rate is high. This may be due to inadequate use of the diagnostic potential.

**Methods:**

We evaluated the misjudgement rate of the aetiology of vaginal complaints. A total of 220 vaginal samples from women with a vaginal complaint were obtained and analysed for numbers of total lactobacilli, H_2_O_2_-producing lactobacilli, total aerobic cell counts and total anaerobic cell counts including bifidobacteria, *Bacteroides *spp., *Prevotella *spp. Additionally, the presence of *Atopobium vaginae*, *Gardnerella vaginalis*, *Candida *spp. and *Trichomonas vaginalis *was evaluated by DNA-hybridisation using the PCR and Affirm VPIII Microbial Identification Test, respectively.

**Results:**

The participating physicians diagnosed Bacterial vaginosis (BV) as origin of discomfort in 80 cases, candidiasis in 109 cases and mixed infections in 8 cases. However, a present BV, defined as lack of H_2_O_2_-lactobacilli, presence of marker organisms, such as *G. vaginalis*, *Bacteroides *spp. or *Atopobium vaginae*, and an elevated pH were identified in only 45 cases of the women examined. *Candida *spp. were detected in 46 cases. Interestingly, an elevated pH corresponded solely to the presence of *Atopobium vaginae*, which was detected in 11 cases.

**Conclusion:**

Errors in the diagnosis of BV and candida vulvovaginitis (CV) were high. Interestingly, the cases of misjudgement of CV (77%) were more numerous than that of BV (61%). The use of Amsel criteria or microscopy did not reduce the number of misinterpretations. The study reveals that the misdiagnosis of vaginal complaints is rather high.

## Backround

The microbiology of the vagina is complex, containing 10^9 ^bacterial colony forming units per gram of secretions and potentially dozens of species. It is mainly dominated by members of the genus *Lactobacillus*, which are capable of H_2_O_2_-production and lactic acid, thereby maintaining the generally acidic vaginal pH. Age, phase of the menstrual cycle, sexual activity, contraceptive choice, pregnancy, presence of necrotic tissue or foreign bodies, and use of hygienic products or antibiotics can disrupt this ecosystem. A disturbed vaginal microbiota is characterised as vaginitis. Vaginitis, whether infectious or not, poses one of the most common problems in gynaecology, and it is one of the main reasons leading women to seek advice from a physician [1]. However, diagnosis and treatment can be elusive, if based on clinical symptoms and the characterisation of vaginal discharge alone [2,3]. This may lead to a lack of relief from symptoms and, because of inadequate treatment, to increased costs for the patient and social securities.

Bacterial vaginosis (BV) and candida vulvovaginitis are responsible for 90% of cases of vaginitis [4]. BV is characterised by the substitution of the regular, lactobacilli dominated microbiota by a complex and abundant flora of strictly or facultatively anaerobic bacteria (*Gardnerella vaginalis*, *Bacteroides *spp., *Prevotella *spp., *Peptostreptococcus *spp., and *Mycoplasma hominis*) [5-8]. Recent studies suggested a correlation between the metronidazole-resistant anaerobe *Atopobium vaginae *and BV [9-12].

Many cases of BV are asymptomatic or present with only malodorous vaginal discharge and no inflammatory complaints. In contrast, CV often correlates with scant discharge and marked inflammatory symptoms (pruritus and soreness) [13] and is characterised by an excessive proliferation of *Candida spp*. in the vaginal flora [14].

Due to the fact that clinical symptoms associated with vaginitis are various, the clinical diagnosis is often deceptive. As defined by Amsel and colleagues [15] a correct diagnosis of BV should comprise at least three of the following criteria: a thin homogeneous white discharge, the presence of "clue cells" on microscopic exam, a pH > 4.5 of vaginal fluid and fishy odour before or after adding 10% KOH (whiff test). Gram-staining of vaginal secretion is also an accepted additional diagnostic test of BV [16-19]. A current CV should be diagnosed by performing saline microscopy in order to look for candidal buds or hyphae. Additionally, the diagnostic panel can comprise culturing, PCR or antibody detection [20-22].

The purpose of this study was to evaluate, whether practitioners truly rely on the recommended criteria for diagnosis of BV [15,18] and if so, whether the use of these criteria leads to a correct diagnosis according to our definition of BV. In addition, we evaluated the accuracy for the diagnosis of CV. In order to do so, we used a combination of culturing methods, DNA hybridisation and PCR for the differentiation of the various vaginal infectious types.

## Methods

### Participants and subjects

220 women diagnosed with relapsing vaginitis by the same physician within a period of three months were included in the study. "Relapsing" was defined as seeking medical help for identical symptoms. The age of the participants ranged from 17 to 65 years. Twelve participants were postmenopausal women. Vaginal samples were sent in by 53 general practitioners and 31 gynaecologists.

### Collection of samples for analysis

All participating physicians used the same test system (VaginalStatus, IFM, Herborn, Germany), which contained two swabs. Using these swabs two vaginal samples were taken from each individual. One swab (pre-weighed) was placed into Portagerm-Amies-Agar transport tubes (bioMérieux) and was used for the culturing of aerobic and anaerobic bacteria. The second swab (Affirm hardwood swab; Becton Dickinson, Heidelberg, Germany) was used for DNA hybridisation and PCR analysis. It was placed into an inactivation medium provided by the supplier (Affirm VPIII; Becton Dickinson and Company, Spaks, USA, [23]). The VaginalStatus-test system was sent to the laboratory for further analysis. In addition, the vaginal pH was determined by litmus paper and documented by the physician.

### Transport of samples

The performance of the Affirm VPIII following transport was evaluated elsewhere [24]. To ensure that the transport did not have any effect on the cultured species, a storage study was performed with vaginal samples from 10 healthy women and 10 women diagnosed with BV. Two vaginal samples were taken from each individual and both were placed in Portagerm-Amies-Agar transport tubes. The first swab was immediately removed from the transport medium and put in 1 ml of phosphate-buffered saline (PBS, pH 7.2)

The solution was vortexed for 5 s and serially diluted (neat to 10^-5^) in PBS, pH 7.2. One ml of each dilution was plated onto enrichment or selective agar media [Columbia blood agar for aerobic bacteria, Schaedler agar for anaerobic bacteria, DIC agar for *Bifidobacterium *spp., CPS agar for *Enterobacteriaceae *and Rogosa agar for *Lactobacillus*spp. (bioMérieux, Nuertingen, Germany)]. The second swab was stored in the transport medium for three days at 25°C, which represents the average temperature during shipment. Following the incubation period, the second swab was processed as described for the first and the results were compared. No significant differences in the cell counts of the investigated genera could be detected. Thus, it was concluded that a shipment of less than three days did not affect the composition of the studied microbiota. Therefore, only samples that took less than 3 days to arrive at the laboratory were included in the study.

### Identification and enumeration of microorganisms

Vaginal samples were plated on selective agar (see above) and subsequently incubated under aerobic or anoxic conditions at 37°C for two days. The bacteria were submitted to Gram staining and the colony morphologies recorded. Additionally, identifications were performed by the API^®^- and VITEK^®^-systems (bioMérieux). All counts were listed as the numbers of log_10 _colony forming units (CFU) per ml of sample.

### Detection of H_2_O_2_-production

Following identification, lactobacilli were tested for hydrogen peroxide production as described previously [25]. Colonies that produced H_2_O_2 _on the agar appeared dark blue. Colonies, which did not produce H_2_O_2_, were colourless.

### DNA-hybridisation

Hybridisation was performed as described by the manufacturer (Affirm VPIII; Becton Dickinson and Company, Sparks, USA). The test system is based on two specific single-stranded nucleic acid probes for each organism as well as on capture probes and colour development probes that are complementary to unique genetic sequences of *Gardnerella vaginalis*, *Candida *spp. and *Trichomonas vaginalis*.

### Detection of Atopobium vaginae

For the detection of *Atopobium vaginae *the PCR-method described by Ferris *et al*. was used [26].

### Evaluation criteria

Within the presented study the following criteria were analysed: Total aerobic and anaerobic cell counts, numbers of H_2_O_2_-producing lactobacilli, bifidobacteria, *Bacteroides *spp., *Prevotella *spp., *Streptococcus agalactiae *and *Staphylococcus *spp. In addition, *Gardnerella vaginalis*, *Candida *spp. or *Trichomonas vaginalis *were detected by DNA-hybridisation (Affirm VPIII) and *Atopobium vaginae *by PCR, respectively. Bacterial vaginosis (BV) was defined as manifested, if the following three criteria were fulfilled: elevated pH, decrease of H_2_O_2_-producing lactobacilli, presence of either *G. vaginalis*, *A. vaginae *or any other anaerobic organism. A positive DNA hybridisation was used as the gold standard for diagnosis of candida vulvovaginitis (CV). All participating physicians completed a questionnaire, whereby the presence of the following symptoms was recorded: itching, burning, odour, redness and discharge. Furthermore, the use of Amsel-criteria, Nugent-score [15,18], microscopy for the identification of fungi and suspected causative of infection were inquired.

### Statistical analysis

All data were compiled and analysed using SPSS (SPSS Inc., Chicago, IL.). *P *values for the difference in speeds of various methods were calculated using Wilcoxon's nonparametric test. The chi-square procedure was used to compare the isolation frequency of bacterial groups between categories of different vaginal pH. Diagnostic accuracy was measured by computing point estimates of the following test properties for each response, using standard methods: sensitivity, specificity, positive and negative predictive values, positive (LR+) and negative likelihood ratios (LR-). The precision of these estimates was evaluated using 95% confidence intervals. We used the kappa (κ) statistic to evaluate chance-corrected agreement between the diagnosis by physicians and our microbiologically based diagnosis. A kappa value of 0 indicates that the observed agreement is the same as that expected by chance. A kappa value of 1 indicates perfect agreement. The following guides were used for interpreting the kappa statistic: a kappa value of < 0.2 indicates poor agreement, 0.21–0.6 fair to moderate agreement, 0.61–0.8 good agreement and 0.81–1.0 very good agreement [27].

## Results

Of the 220 vaginal samples obtained only 197 were included into the study, as 23 samples did not fulfil all evaluation criteria. No pH was recorded in 12 samples, no admission diagnosis was given in 10 samples, and one sample contained only the swab for DNA-hybridisation.

A comparison of frequencies of detection and mean counts of the major genera comprising the vaginal microbiota in relation to the recorded vaginal pH is shown in Table 1. No significant differences in the frequency of isolation of the various lactobacilli types (H_2_O_2_-producers and non-producers) could be observed between women with a normal pH and women with an elevated pH, respectively. However, cell counts were decreased by one order of magnitude between the two groups. A lower cell count was recorded for the group with an elevated pH. Interestingly, high rates of H_2_O_2_-producing lactobacilli (> 10^8^CFU/ml) were significantly (*P *< 0.05) more often reduced in women who were recorded with an elevated vaginal pH. In several samples positive for H_2_O_2_-producing lactobacilli, colonies which appeared light blue were seen, but their amount was always less than the number of dark blue colonies.

**Table 1:** Frequency of isolation and cell numbers of organisms from vaginal samples related to vaginal pH

	Determined vaginal pH			
		
Organisms	< 4.5		≥ 4.5	
		
	Frequency of isolation^1^	No. of organisms^2^	Frequency of isolation^1^	No. of organisms^2^
**Aerobic flora**				
Total aerobes^3^	109/115	7.20 ± 0.37	65/82	6.65 ± 1.57
*Lactobacillus *spp.	107/115	7.69 ± 1.61	61/82	6.58 ± 1.88
H_2_O_2_-positive lactobacilli	102/115	7.77 ± 1.53	56/82	6.46 ± 1.87
H_2_O_2_-positive lactobacilli (>10^8 ^CFU/ml)	64/115	8.72 ± 0.43	26/82^6^	8.42 ± 0.65
**Anaerobic flora**				
Total anaerobic isolates	5/115	7.71 ± 0.32	9/82	8.06 ± 0.12
*Bifidobacterium *spp.	3/115	8.03 ± 0.42	5/82	7.94 ± 0.64
*Bacteroides *spp.	2/115	7.39 ± 0.09	4/82	8.19 ± 0.53
*Prevotella *spp.	0/115	n.d.^4^	0/82	n.d.^4^
**Molecular detection**				
*Gardnerella vaginalis*	26/115	-^5^	25/82	-^5^
*Candida *spp.	28/115	-^5^	18/82	-^5^
*Trichomonas vaginalis*	0/115	-^5^	0/82	-^5^
*Atopobium vaginae*	0/115	-^5^	11/82^6^	-^5^

^1 ^Values are numbers of observations/numbers of subjects

^2 ^Values are log_10 _CFU/ml sample ± standard deviations

^3 ^Including *Coliform *spp., *Staphylococcus *spp. and *Staphylococcus *spp.

^4 ^not detected

^5 ^no cell counts can be given due to the used methods (hybridisation and PCR, respectively)

^6 ^P < 0.05 when compared to values for normal pH using the chi-square procedure

In 45 cases an elevated pH was accompanied by a decrease in the frequency of H_2_O_2_-lactobacilli as well as by the presence of anaerobes, and was accordingly classified as bacterial vaginitis (BV). *Candida *spp. were detected in 46 cases and mixed infections in one case, whereas in 105 cases no sign of an infectious vaginitis could be identified. The mean counts of total aerobes, anaerobes, *Staphylococcus agalactiae*, *Streptococcus *spp., *Bacteroides *spp., and bifidobacteria were similar in women with a normal pH and women with an elevated pH. However, the amount of *Bacteroides*spp. was elevated by one order of magnitude in women with an elevated pH. No other anaerobic bacteria were identified. Furthermore, no significant differences in the presence of *Gardnerella vaginalis, Candida *spp. and *Trichomonas vaginalis *were detected. However, there was a significant correlation between *Atopobium vaginae *and an elevated pH. Interestingly, *A. vaginae *was always detected in combination with *G. vaginalis*. Within the study group no correlations between pre- and post-menopausal women, vaginal complaints and the microbiota could be drawn.

The results of the 197 questionnaires are summarised in Tables 2 and 3. Itching and burning were the most common symptoms reported, followed by discharge, redness, and odour (Table 2). None of the reported symptoms could be associated with the presence of a specific organism (*P *> 0.05; data not shown). The diagnosis of the participating physicians was BV in 80 cases, candida vulvovaginitis (CV) in 109 cases, and mixed infection in 8 cases (Table 3). Amsel-criteria were used in 54 out of 80 cases for the diagnosis of BV, microscopy in 60 out of 109 cases for the diagnosis of candida vulvovaginitis, and both were used in four out of eight cases for mixed infections (Table 3). Both, Amsel criteria and microscopy, were predominantly but not exclusively used by gynaecologists (data not shown). Not a single physician based his diagnosis on Nugent-score [18].

**Table 2:** Incidence and frequency of reported complaints

Complain	Incidence ^1^	Frequency ^2 ^(n = 197)
Itching	125	63.45
Burning	114	57.87
Redness	74	37.56
Discharge	93	47.21
Odour	35	17.77

^1 ^multiple selections were possible

^2 ^% of total

**Table 3:** Correctness of admission diagnosis

			Amsel criteria and/or microscopy^1 ^was used			
Diagnosis of physicians	Diagnosis was				Diagnosis was	
			
	correct	incorrect	yes	no	correct	incorrect
Bacterial vaginosis (n = 80)	31 (38.8)^2^	49 (61.3)	54 (67.5)	26 (32.5)	20 (37)^3^	34 (63)
Candida infection (n = 109)	25 (22.9)	84 (77.1)	60 (55)	49 (45)	15 (25)	45 (75)
Mixed infection (n = 8)	1 (12.5)	7 (87.5)	4 (50)	4 (50)	1 (25)	3 (75)

^1 ^Microscopy refers to saline wet mount

^2 ^% of total

^3 ^% of cases where Amsel criteria or microscopy were used

The diagnosis of BV by physicians agreed only fairly with our diagnosis (κ = 0.29; 95% CI 0.16, 0.42). There was an even poorer agreement for the diagnosis of candidiasis (κ = -0.01; 95% CI -0.12, 0.10). Regardless of the implementation of the various recommended criteria for evaluation of vaginitis [21,28], the error of judgement for BV, CV or a mixed infection was high (Table 3). The level of misdiagnosis exceeded 70%. The misjudgement of a mixed infection was highest (87%), followed by CV (77%) and a manifested BV (>61%). The use of the Amsel criteria for the detection of BV or microscopy for the detection of *Candida *spp. did not enhance the diagnostic correctness (Table 3). Compared to the detection of a pathological vaginal colonisation as reference standard, which was based on our microbial verification of the pathologic agent or altered vaginal microbiota, the diagnostic classification by physicians was of relatively minor accuracy. Sensitivity and specificity (95% confidence interval), respectively, were 68.9% (55–82) and 67.8% (60–75) for the diagnosis BV, whereas only 54.3% (40–69) and 44.4% (36–52) for the diagnosis of a CV. Positive and negative predictive values as well as likelihood ratios (LR) of a positive and negative result were 38.8% (28–50), 88% (80–93), 2.1 (1.6–2.9) and 0.46 (0.29–0.71), respectively, for the diagnosis BV. The corresponding values for the diagnosis of a CV were 22.9% (15–31), 76.1 (65–84), 0.97 (0.72–1.31) and 1.03 (0.72–1.47), respectively. From these values the diagnostic odds ratios were calculated for the diagnosis of BV and Candidiasis with values of 4.66 (95% CI: 0.51, 2.16) and 0.95 (95% CI: 0.49, 1.84), respectively, indicating again that the diagnosis of a CV by physicians does not discriminate between diseased and not diseased.

## Discussion

Studies showed that bacterial vaginosis (BV) resulted from an imbalance of the normal vaginal flora, with an overgrowth of gram-negative anaerobes and a reduction in lactobacilli, especially those that produced H_2_O_2 _[29-32]. Candida vulvovaginitis (CV) was primarily associated with *Candida albicans*, but other species are emerging [33,34]. The standard diagnosis for BV should enclose the Amsel-criteria [28]. For the identification of fungal infections, culturing or microscopy was recommended [21].

However, error in diagnosis was rather high within the group of surveyed physicians, no matter whether they used the recommended techniques or not. The establishment of a wrong diagnosis was highest for mixed infections followed by CV and BV. This fact may be explained by an inadequate use of microscopy, as microscopy is the key technique in recognition of a vaginal infection. Microscopy without specific staining generally leaves room for interpretation. In example, none of the participating physicians detected *A. vaginae *under the microscope or interpreted the presence of small elliptical cocci as conspicuous. When asked in retrospect, none of the participating physicians was aware of the existence of this species. Taken into account that *A. vaginae *is resistant to metronidazol, a common antibiotic given for the treatment of BV, a proof of absence of this organism should be mandatory at least in women with recurring vaginal complaints.

According to its frequent citing in scientific publications the Nugent-score is well established for the diagnosis of BV [7,17,35,36]. However, the herein presented results suggest that it is not commonly used on a day to day basis within the practicing community of physicians.

In recent years, the capability of lactobacilli to protect against vaginal infections by altering vaginal pH through the production of metabolic end-products including lactic acid, and by their ability to produce hydrogen peroxide (H_2_O_2_) has been widely recognised [31,32,40-42]. If those lactobacilli, which predominantly produce H_2_O_2 _are missing or underrepresented, serious problems including BV [42,43], recurrent urinary tract infections [32] or even an increased risk of pre-term delivery are likely to occur [30,44]. Interestingly, in over 50% of cases within our study, no association of complaints with BV or candida vulvovaginitis could be drawn. However, within this group of patients, low cell counts of H_2_O_2_-producing lactobacilli were observed. Lactobacilli, especially those capable of H_2_O_2_-production, are responsible for the maintenance of the normal vaginal ecosystem [29,31,45]. Therefore, in cases where lactobacilli cell counts are low it may be favourable to supplement the vaginal flora with lactobacilli to help restore the usual conditions.

Concerning the cases of misdiagnosed fungal infections, which occurred more frequently than cases of misdiagnosed BV, our results are in accordance with the literature [2,22]. Because microscopic evaluation requires special diagnostic skills not available to all practitioners, misjudgement is common, therapy is frequently empirical and the relapse rate is high [20]. In our study, the recognition or the exclusion of the diagnosis candidiasis by surveyed physicians was nearly as likely in patients with the disease as in patients without the disease. Physicians diagnosed 54.3% of all candida infections correctly, but had a false positive CV diagnosis for 55.6% of all not infected cases. This is also demonstrated by the LR values close to 1, with which tests cannot help a clinician to rule in or rule out the target disease. Therefore, the diagnosis of a CV by physicians was not better than those expected by chance as judged by the positive and negative likelihood ratios. In one recent study, the sensitivity of microscopy for diagnosis of candida vulvovaginitis was only 22% [46]. Therefore, a more reliable test system, like the herein implemented hybridisation system (Affirm VPIII), which detects several *Candida *spp. (*C. albicans*, *C. glabrata*, *C. kefyr*, *C. krusei*, *C. parapsilosis and C. tropicalis*) is necessary.

Our results are in accordance with a recent study, which concluded that the clinical diagnosis of vaginal infections (history and physical examination, vaginal pH, wet mount microscopy, whiff test) is inadequate and should be confirmed by microbiological testing [47]. This was also reported by Anderson *et al*., who evaluated the role of clinical examinations in published articles from 1966 to 2003 [48]. The poor performance of individual symptoms and signs left one third of women with vaginal complaints without an adequate diagnosis. In general, laboratory tests should be more emphasized in the diagnosis of vaginal complaints.

## Conclusion

It becomes evident from this study that the misjudgement of vaginal complaints is rather high and that the so called "gold-standards" for the diagnosis of vaginal infections are not widely and/or correctly used. Ultimately, a correct diagnosis is not yielded; it is rather throwing a dice for the diagnosis.

## Competing interests

Herewith, the authors declare that they obtain, except for DT, funding by the Institute for Microecology.

## Authors' contributions

AS, KR and VR planned and carried out the design of the study. AS and KR participated in laboratory studies and interpretation of the data. AS wrote the first draft of the manuscript. DT carried out the statistics. All authors had intellectual contribution, and all read and approved of the final manuscript.
